# Supplementary material for: Facile Synthesis of Zn-Co-S Nanostrip Cluster Arrays on Ni Foam for High-Performance Hybrid Supercapacitors
Source: Nanomaterials (Basel). 2021 Nov 26;11(12):3209. doi: 10.3390/nano11123209 (PMC8706522; doi:10.3390/nano11123209)
Supplement: Supplementary file 1 [file nanomaterials-11-03209-s001.zip › nanomaterials-1415531-supplementary.pdf]

Supplementary Information

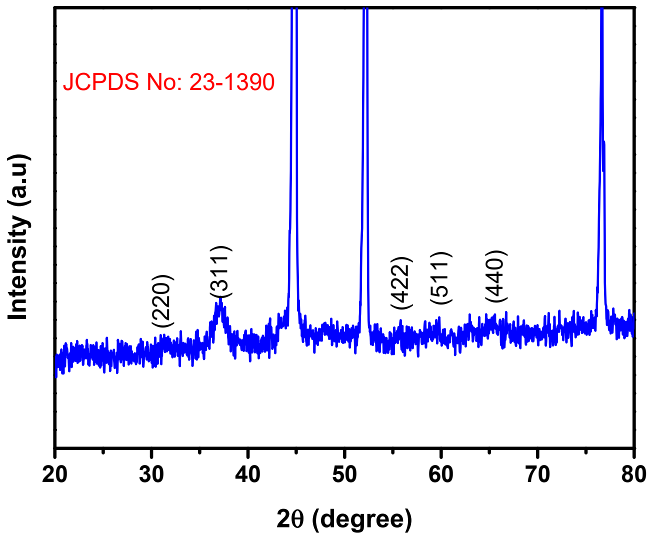

Figure S1. XRD pattern of the as-prepared $ZnCo_2O_4$.

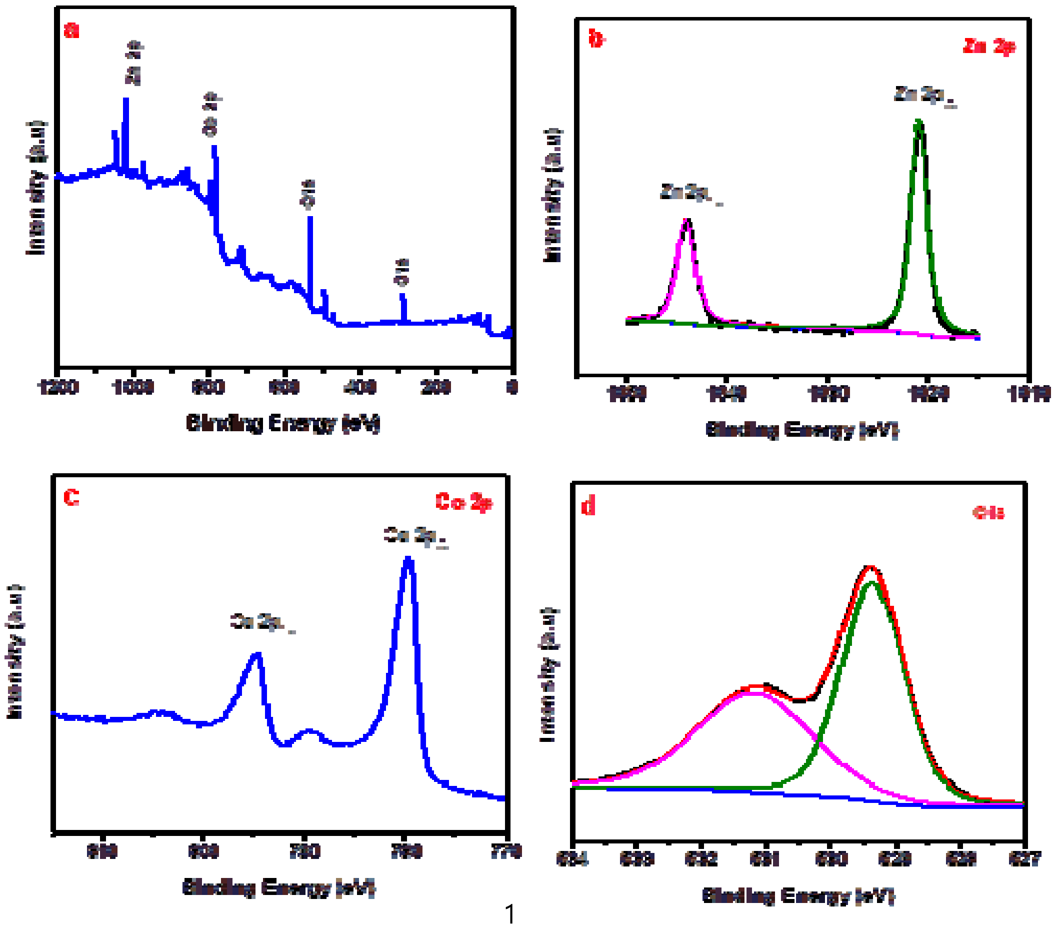

Figure S2. (**a**) XPS survey spectrum and (**b**) Zn 2p, (**c**) Co 2p, and (**d**) O 1S high-resolution spectra of $ZnCo_2O_4$.

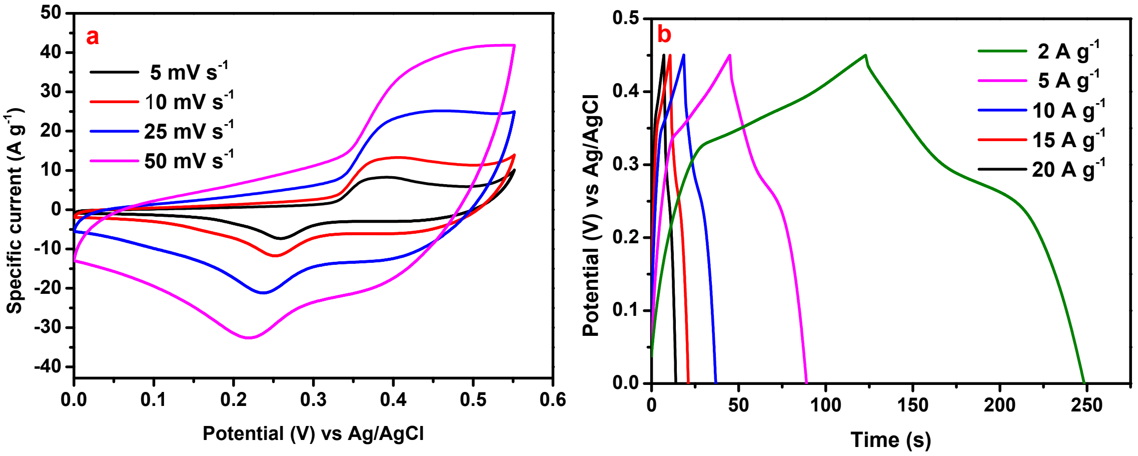

Figure S3. (**a**) CV curve at different scan rates and (**b**) the charge-discharge curves of the $ZnCo_2O_4$ electrode.

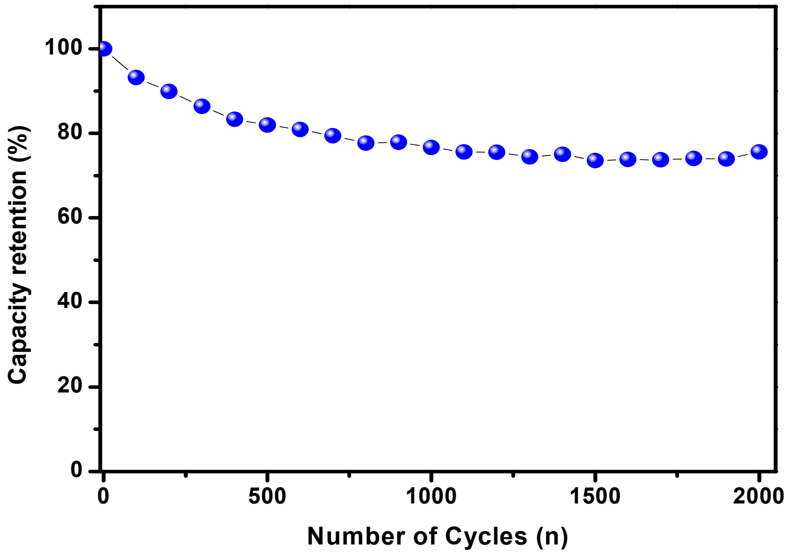

Figure S4. Cycling stability curve of the Zn-Co-S electrode measured at 20 A g^{-1}.

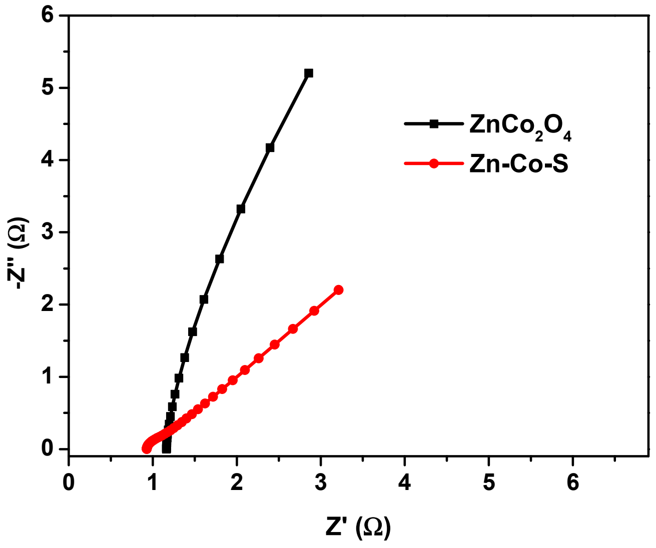

Figure S5. Impedance plots of the $ZnCo_2O_4$ and Zn-Co-S electrodes.

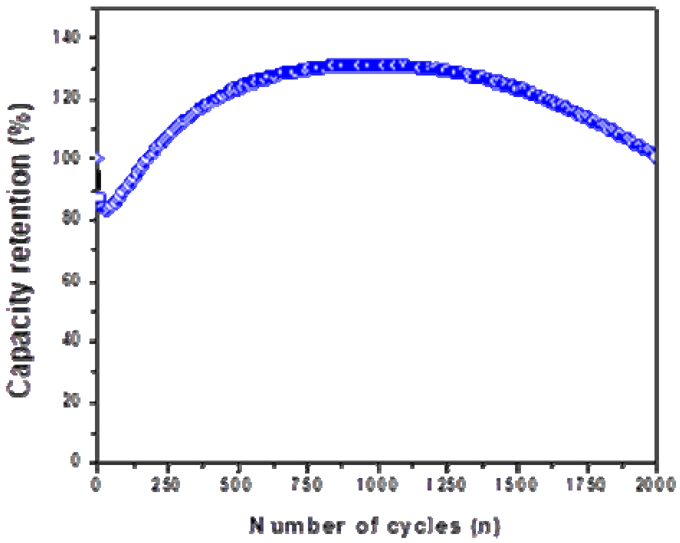

Figure S6. Cycling stability curve of the Zn-Co-S//AC asymmetric hybrid supercapacitor device.

Performance comparison Tables

Table S1. Comparison of electrochemical performance of Zn-Co-S based supercapacitor

Electrode material	C_s [+] (F g^{-1})	ED (Wh kg^{-1})	PD (W kg^{-1})	Cycling stability	Ref.
$Zn_xCo_{1-x}S$//AC	486 (2 A g^{-1})	14.0	450	NA	[1]
CoSx/C//PCNFs	497 (0.5 A g^{-1})	15.0	413	80% (2000 cy)	[2]
ZnCoS//rGO	1134 (1 A g^{-1})	17.7	435	84% (5000 cy)	[3]
PPY/GO/ZnO// PPY/GO/ZnO	NA	10.6	258	74% (1000 cy)	[4]
ZnO/GNR// LRGONR	450 (5 mV s^{-1})	9.4	1187	97% (5000 cy)	[5]
rGO/CoS_2//AC	636 (1 A g^{-1})	13.8	824	NA	[6]
Zn-Co-S//AC	1840 (2 A g^{-1})	19.0	514	100% (2000 cy)	**This work**

C_s**[+]** = Specific capacitance of a positive electrode
AC = activated carbon, PCNF = porous carbon nanofibers, cy = cycle
PPY = Polypyrrole, LRGONR = lacey reduced graphene oxide nanoribbons

References

1. Yang, J.; Zhang, Y.; Sun, C.; Guo, G.; Sun, W.; Huang, W.; Yan, Q.; Dong, X. Controlled synthesis of zinc cobalt sulfide nanostructures in oil phase and their potential applications in electrochemical energy storage. *J. Mater. Chem. A* **2015**, *3*, 11462–11470, doi:10.1039/c5ta01739d.

2. Liu, Y.; Zhou, J.; Fu, W.; Zhang, P.; Pan, X.; Xie, E. In situ synthesis of CoSx@carbon core-shell nanospheres decorated in carbon nanofibers for capacitor electrodes with superior rate and cycling performances. *Carbon* **2017**, *114*, 187–197, doi:10.1016/j.carbon.2016.12.018.

3. Zhang, Y.; Cao, N.; Szunerits, S.; Addad, A.; Roussel, P.; Boukherroub, R. Fabrication of ZnCoS nanomaterial for high energy flexible asymmetric supercapacitors. *Chem. Eng. J.* **2019**, *374*, 347–358, doi:10.1016/J.CEJ.2019.05.181.

4. Chee, W.K.; Lim, H.N.; Harrison, I.; Chong, K.F.; Zainal, Z.; Ng, C.H.; Huang, N.M. Performance of Flexible and Binderless Polypyrrole/Graphene Oxide/Zinc Oxide Supercapacitor Electrode in a Symmetrical Two-Electrode Configuration. *Electrochim. Acta* **2015**, *157*, 88–94,

doi:10.1016/J.ELECTACTA.2015.01.080.

5. Sahu, V.; Goel, S.; Sharma, R.K.; Singh, G. Zinc oxide nanoring embedded lacey graphene nanoribbons in symmetric/asymmetric electrochemical capacitive energy storage. *Nanoscale* **2015**, *7*, 20642, doi:10.1039/c5nr06083d.

6. Chen, Q.; Cai, D.; Zhan, H. Construction of reduced graphene oxide nanofibers and cobalt sulfide nanocomposite for pseudocapacitors with enhanced performance. *J. Alloys Compd.* **2017**, *706*, 126–132, doi:10.1016/J.JALLCOM.2017.02.189.
